# Breastfeeding *versus* free distribution of infant formulas by the Public Health System

**DOI:** 10.31744/einstein_journal/2021AO6451

**Published:** 2021-11-03

**Authors:** Flávia Galvão Cândido, Brunnella Alcântara Chagas de Freitas, Rita de Cássia Santos Soares, Jersica Martins Bittencourt, Daniela Neves Ribeiro, Dayane de Castro Morais, Camilla de Freitas Niquine, Sarah Aparecida Vieira Ribeiro, Raquel Maria Amaral Araújo, Bruna Romano Zucchetto, Taimã de Castro Carvalho, Isabela Carvalho Rezende

**Affiliations:** 1 Centro Estadual de Atenção Especializada ViçosaMG Brazil Centro Estadual de Atenção Especializada, Viçosa, MG, Brazil.; 2 Universidade Federal de Viçosa ViçosaMG Brazil Universidade Federal de Viçosa, Viçosa, MG, Brazil.

**Keywords:** Breast feeding, Infant formula, Prescriptions, Unified Health System

## Abstract

**Objective:**

To characterize the situation of breastfeeding and the adequacy of prescription of infant formulas to infants assisted by a secondary care program of the Public Health System.

**Methods:**

This is a cross-sectional study with analysis of medical records of 350 infants from zero to 6 months, followed up between February to April 2019.

**Results:**

The possibility of breastfeeding was present in 97.0% of mothers and no infant presented an acceptable medical condition for proscription of breastfeeding. Despite this, only 47.2% of cases were on exclusive breastfeeding before being referred to the program. Regarding the reasons for the introduction of infant formulas, complementation to breast milk was the most present (75.8%), followed by mothers returning to the job market (20.1%). The general rates of inadequacy of those prescribed were 65% before arriving at the program, increasing to 69% (standard formulas) and 80% (formulas for special purposes) during follow-up.

**Conclusion:**

The low rate of exclusive breastfeeding and the indiscriminate prescription of infant formulas are a concern for damage to maternal-child healthcare and sound finances of the Public Health System.

## INTRODUCTION

Breast milk is undoubtedly the best food for newborns and should be maintained exclusively until 6 months of life.^( 1 )^ It is estimated that if breastfeeding were extended to almost universal levels, about 820 thousand children's lives would be saved every year.^( 2 )^ Globally, only 40% of children under 6 months of age receive exclusive breastfeeding (EBF). In Brazil, despite the improvement in breastfeeding indicators, surveys show a tendency toward stabilization of rates in recent years, with only 36.6% of infants receiving EBF by the age of 6 months.^( 3 )^

The Ministry of Health has adopted important initiatives aimed to improve national EBF indicators.^( 1 – 4 )^ However, concomitant with these efforts and justified by the need to protect infants at nutritional risk, many programs have been developed in several Brazilian regions to promote the free distribution of infant formulas as breast milk substitutes, by the Brazilian Public Health System (SUS - *Sistema Único de Saúde* ).^( 5 )^

Infant formulas are developed to resemble breast milk and can be prescribed for very specific medical and/or nutritional conditions, most often for a limited period.^( 6 )^ However, the composition of infant formulas does not match the physiological and nutritional properties of human milk, and negatively impacts the success and duration of breastfeeding.^( 5 )^ The Ministry of Health highlights as “absolutely condemnable the large-scale distribution of these products, especially in health services”.^( 5 )^ Excluding cases in which there are acceptable medical reasons for the use of infant milks or formulas, the prescription of these products for infants who do not need these foods should be considered inappropriate.^( 7 )^

Another aspect of the distribution to be considered refers to the economic repercussions of use of infant formulas. According to federal law 8.080/1990, food is characterized as one of the conditioning factors of health (Article 3, *caput* ), and establishes nutritional surveillance and food guidance (Article 6) as specific attributions of SUS. Thus, in cases in which food assumes a drug status, as is the case of infant formulas, the public sector is responsible for providing diets, according to the principles and rules of SUS. According to Article 6, “Education, health, food, work, housing, transportation, leisure, security, social security, protection of motherhood and childhood, and assistance to the destitute are social rights, in the form of this Constitution”.^( 8 )^

Given the considerable number of prescriptions for infant formulas without well-established indications, it has become urgent to establish criteria to rationalize access and propose a feasible, equitable, and equal flow for their dispensing, optimizing public resources spent on the purchase of infant formulas.^( 5 )^

## OBJECTIVE

To characterize the breastfeeding situation and the adequacy of infant formula prescriptions to infants assisted by a Secondary Care program of the Brazilian Public Health System.

## METHODS

### Design

This is an exploratory quantitative cross-sectional study conducted at the *Centro* *Estadual de Atenção Especializada* (CEAE) [State Center for Specialized Care], in the city of Viçosa (MG). Data were obtained from medical records of patients aged zero and 6 months referred to CEAE, assisted by the *Leite para Todos* Program between February to April 2019.

Patients were excluded if they did not meet the inclusion criteria for infants at the CEAE described in table 1 , if there was a lack of data in the medical record about breastfeeding, if they were not seen by the nutrition service of the center, if there was an absence of reliable retrospective data in the medical records, if the mothers were adolescents (aged < 16 years), and if they had their first nutrition visit and/or started follow-up after the established period.

**Table 1:** Criteria for inclusion of neonatology/pediatrics patients

Neonatology
Prematurity ≤34 weeks or birth weight ≤1,800g
Premature or non-premature newborns who had acute fetal distress: Apgar score <+7 at the fifth minute of life
Discharged newborns from neonatal progressive care units (neonatal intensive care unit, conventional intermediate care unit, kangaroo intermediate care unit)
Perinatal transmission of infectious diseases: HIV, TORCHS syndrome, and Zika virus
Diseases classified as rare that require management by a multiprofessional team, including the need for specific medication or diet
Premature babies with chronic lung disease
Newborns with neuropsychomotor developmental abnormalities
Newborns with chronic hematological abnormalities

Pediatrics
Children discharged from the pediatric ICU
Children with chronic gastrointestinal diseases with nutritional repercussions (chronic diarrhea, celiac disease, biliary atresia, cirrhosis, food allergies, etc.)
Children with congenital immunodeficiency disorders
Children with chronic renal disorders (repeated UTI, nephrosis, hematuria, etc.)
Children with oncological problems
Children with psychiatric disorders and/or ADHD
Children with diabetes (first consultation, the others in Primary Care)
Children with congenital heart diseases and hypertension
Diseases classified as rare that require management by a multiprofessional team, including the use of medication and/or specific diet

TORCHS: toxoplasmosis, rubella, cytomegalovirus, herpes simplex, syphilis; ICU: intensive care unit; UTI: urinary tract infection; ADHD: attention-deficit hyperactivity disorder.

### Data collection spreadsheets

During the development of the data collection spreadsheet, 20 questionnaires were randomly selected to be evaluated regarding type, quality, and relevance of the information recorded relative to the program proposal. From then on, according to the experiences of the professionals/authors and review of the medical records included in the analysis, the important questions to be answered were selected for the characterization of the problem and the development of solutions. The selection of socioeconomic and demographic, general health, and nutrition variables was based on their ability to answer the questions and allow preparation of solutions, as well as on their epidemiological importance, as indicated by the scientific literature on the subject.^( 9 )^

The socioeconomic and demographic variables were sex, city of origin, maternal education level, family income, and unit of referral to the CEAE. The general health variables were number of previous pregnancies, duration of nutritional monitoring in the CEAE, and presence of maternal or infant conditions/diseases that made breastfeeding unfeasible or compromised. The nutrition variables were type of breastfeeding before and during follow-up, infant formula prescription (standard or special purpose), adequacy of formula prescription, and duration of breastfeeding after formula prescription.

### Data analysis

The Acceptable Medical Reasons for the Use of Breastmilk Substitutes (HIV positive, substance abuse, on chemotherapy, use of medications and alcohol abuse) were used to describe the conditions and diseases that made breastfeeding unfeasible or compromised, as well as to assess the adequacy of infant formulas prescriptions.^( 7 )^ The choice of this document as assessment criterion was due to its wide acceptance by health professionals and managers, and by its objective contents, which enable non-subjective assessment. The use of infant formulas due to changes in the mother or baby's routine, such as attending daycare centers or the mother returning to the job market, was also considered inadequate, since it is not included in the document and because of the possibility of extracting milk to be offered and/or of mothers going to daycare centers to breastfeed.

The analysis of questionnaires was carried out by four previously trained independent evaluators. The doubtful cases were separated and discussed together, to standardize the analysis as much as possible. The absence of consensus was interpreted as unavailable data.

Chronological age in patients born at term (37 to 42 weeks gestation) and corrected age (CA) in patients born prematurely (less than 37 weeks gestation) were adopted to determine the follow-up period of nutritional progression (zero to six months) and the breastfeeding period.^( 10 )^ In cases of premature patients exclusively using infant formula, the evaluation period was carried out until the introduction of complementary feeding, due to the possibility of early introduction of complementary feeding in these cases, never exceeding the lower limit of 4 months of CA.^( 2 )^

Discontinuation of breastfeeding was considered in the following cases: the mother stopped breastfeeding, the infants were using only infant formula, and/or breastfeeding was no longer mentioned in the patient's nutritional progression notes. In cases of discharge at the CEAE by infants on EBF aged less than 6 months, who were properly oriented and encouraged to maintain EBF, continuation of EBF until 6 months was taken into account.

Those whose babies were referred in exclusive use of infant formula and whose records did not contain precise information on maternal lactation conditions were considered missing data on the possibility of breastfeeding by the mother. Infants able to breastfeed were those referred to on EBF or mixed (breastfeeding plus infant formula), with no description of infant conditions that made lactation impossible, or in cases whose conditions were corrected before referral.

Only qualitative aspects were adopted for the evaluation of the adequacy of infant formula prescribing. Special infant formulas were considered as any formulation that was not standard or the starting formulation (type 1 formulas).

The prescriptions that did not meet the acceptable medical reasons for prescribing infant formula, plus other conditions/diseases with potential to hinder breastfeeding, such as scabies, mastitis, poor acceptance of breastfeeding, low milk production, maternal death, and/or impossibility of living with the mother, were considered inappropriate. The adequacy and inadequacy of the prescriptions were assessed at two different timepoints: at the time of referral to the CEAE, and during follow-up at the center. The prescription of hypercaloric formulas was considered appropriate only in cases of infant conditions requiring fluid restriction.

For the calculation of the economic repercussions, the average quantities of formulas recommended by the manufacturer to infants from zero to 6 months and the reported duration for breastfeeding were used as bases, in addition to the average values of standard or special-purpose formulas available in a large retail network. The amounts of formula recommended to meet the infant's needs were entirely converted into monetary values for infants using formula exclusively. For infants on complementary infant formula, the conversion was performed to reach 50% of requirements. Calculations were made according to the type of formula used for each infant. Only those cases in which the prescription was considered inadequate, according to study criteria, were considered in the calculation of the negative economic repercussions.

After collection, data were tabulated on an Excel spreadsheet and statistical analysis was conducted with the help of the (SPSS) software, version 17.0 (SPSS Inc., Chicago, United States).

The variables were tested for normality of data distribution by the Kolmogorov-Smirnov test, and described in absolute and relative frequency. The Mann-Whitney test was performed to compare the data regarding the time of breastfeeding. Spearman's correlation analysis was used for continuous variables. For all these analyses, an alpha of 5% was adopted.

This study was approved by the Research Ethics Committee, CAAE: 16796619.1.0000.5153, and opinion 3.515.075.

## RESULTS

**Figure 1:**
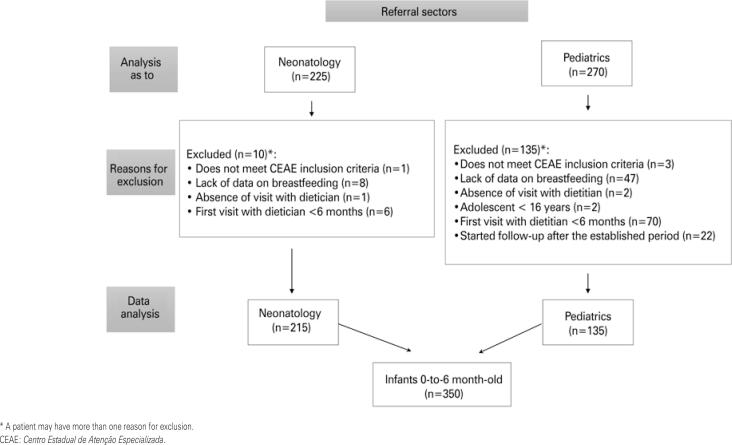
Sample flowchart according to the referral sector and the stages of problem characterization

The total number of records evaluated was 495, and 350 were included based on the selection criteria adopted ( Figure 1 ). The possibility of breastfeeding was present in 97% of the mothers evaluated, and no infant presented a medical condition acceptable for the proscription of breastfeeding. However, only 47.2% of cases were on EBF before referral to the CEAE, with a reduction of this rate to only 28.0% during follow-up at the CEAE. Only 10.9% of neonates who arrived at the CEAE using complementary infant formula were initiated on EBF; 41.1% remained with the complementary use of infant formula, and 48.0% began to use infant formula exclusively.

It was found that 92% (n=149) of patients used infant formula before referral to the CEAE, and continued to use it after follow-up at the center, while 54% (n=94) of those who did not use before the beginning of follow-up continued without this prescription. Thus, there was an association between the use of infant formula prior to referral to the CEAE and continuity of the practice after follow-up at the center, showing the early introduction of formula makes it difficult to resume EBF. Among the records that contained information about the reason for introducing infant formula (n=124), the complement to breast milk was the most common (75.8%), followed by the mother's return to work (20.1%).

Breastfeeding had no significant association with the variables parity, income, and education level. The variables twin pregnancy, parity, income, and education level had no effect on breastfeeding time. Breastfeeding time had a median of 120 days (zero to 1,260 days), whereas the follow-up time at the center was 270 days (15 to 1,740 days). There was a positive correlation between these variables (r=0.176), indicating the increase in the time of follow-up at the center caused an increase in the breastfeeding time of the children, but twins were negatively associated with EBF, as shown in table 2 .

**Table 2:** Relation between exclusive breastfeeding and physiological and socioeconomic characteristics of the mothers evaluated

Variables		Exclusive breastfeeding		p value *
		Yes	No	
Twin pregnancy				
	Yes	13 (30.2)	30 (69.8)	0.015
	No	149 (50.0)	149 (50.0)	
Parity				
	Multiparous	74 (43.0)	98 (57.0)	0.74
	Primiparous	85 (52.8)	76 (47.2)	
Income, minimum wages †				
	≤3	87 (44.6)	108 (55.4)	0.882
	>3	13 (52.0)	12 (48.0)	
Education level				
	≤Incomplete high school	47 (42.7)	63 (57.3)	0.346
	≥Complete high school	52 (43.7)	67 (56.3)	

Results expressed as n (%).

* Pearson's test χ^2^.

† Minimum [monthly] wage considering R$ 1.006,00.

The projections of the estimated economic repercussions of costs with the acquisition of infant and special formulas that could be avoided were R$ 80.814,00 and R$ 55.942,14, respectively. The total cost estimates with special formulas were R$ 85.427,12 to serve 51 infants, while the costs with standard formulas were R$ 110.568,82 to serve 187 infants. Due to the great inadequacy in the prescription of infant formulas, the costs considered avoidable exceeded those needed by 130.9% for all formulas, by 171.6% for standard formula, and by 89.7% for special formulas.

## DISCUSSION

This study showed low rates of EBF, as well as excessive prescription and free distribution of infant formulas in infants less than 6 months of age assisted by a SUS Secondary Care program. Exclusive breastfeeding rates are even lower than the national average (28.0% *versus* 36.6%), which is already considered low by reputable international agencies.^( 3 )^ It is known that breastmilk transfers specific immunological protection from the mother to her child during lactation,^( 11 )^ and is able to benefit the intestinal function and protect the child against pneumonia, ear infections, and allergies, in addition to providing better development of the nervous system.^( 1 , 12 )^

The low EBF rates observed in this study occurred in the absence of restrictive and impeding factors linked to the mother and infant in most of the sample. The only acceptable medical reason linked to the infant that, in fact, implies the impossibility of consuming breastmilk is the presence of inborn errors of metabolism, such as galactosemia and maple syrup urine disease (MSUD),^( 11 )^ which did not occur in any patient evaluated.

The use of infant formulas has been negatively associated with higher prevalence of obesity, diabetes, hypertension, and several types of cancer in adulthood, and breastfeeding is considered a protective factor, in addition to being related to early weaning.^( 2 , 13 )^ Excluding cases in which there are acceptable medical reasons for the use of infant milks or formulas,^( 7 )^ the prescription of these products for children who do not need these foods should be considered inappropriate.

The complementary use of infant formula should be carefully evaluated, since the prescription of breastmilk substitutes at maternity hospitals may encourage mothers to do the same when they return home, believing that they are incapable of producing enough milk for the newborn.^( 14 )^

The results presented highlighted the urgent need to evaluate the current policy of prescription and distribution of infant formulas, which seems to be excessive and inconsistent with national policies to promote breastfeeding. Still, they demonstrated the need for a better liaison with other levels of care (primary and tertiary), since the problem was evidenced even before the patient's arrival for care. Such results, in addition to being a warning sign, due to health issues, also have relevant financial implications for public management.

The Secondary Care unit studied adopts a policy of free distribution of infant formulas upon presentation of a prescription filled out by a dietitian. The release is made by the registered municipalities, a situation similar to that of most Brazilian secondary care units. The calculations in this study were performed to meet the demand of the prescriptions made only for infants who had active medical records during February to April 2019, considering the nutritional needs only from zero to 6 months of age.

According to the estimates of this research, keeping a child on exclusive artificial feeding with standard formulas from 0 to 6 months of age can cost, on average, R$ 1.000,00 for the municipality for the period of 6 months. This amount can increase to approximately R$ 9.800,00 in the same time interval, if the prescription is made for specialized formulas. Since the secondary care units receive new patients throughout the year and it is usual the maintenance of infant formulas until at least 12 months of age, the annual projections can be much higher. Considering the importance of the rational use of public resources, for their limited nature, the consistent prescription of infant formulas assumes the character of public responsibility, by allowing the allocation of scarce resources to those who really need them.

**Figure 2:**
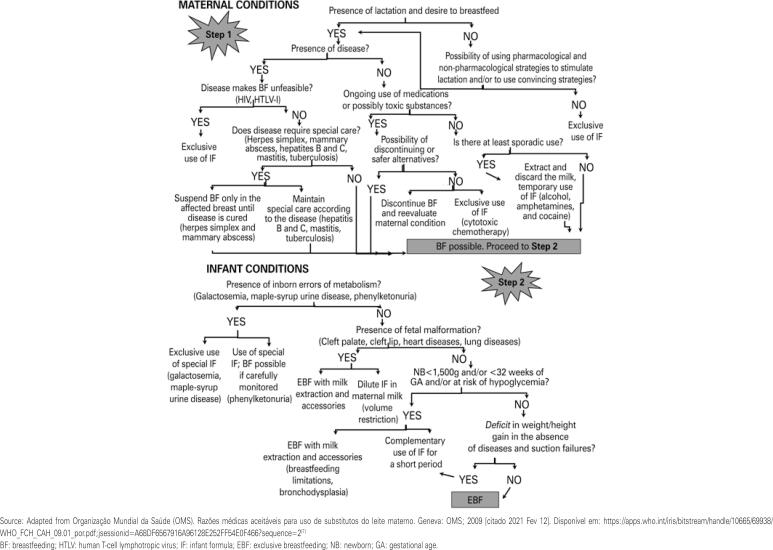
Decision flowchart for prescribing infant formulas to infants from zero to 6 months

Based on the results of this study, a flowchart was developed to help decision making regarding the prescription of infant formula, based on the recommendations of the World Health Organization (WHO),^( 7 )^ which can be used by health professionals, such as pediatricians and dietitians, during the care of infants. Through the evaluation of maternal and child conditions, this tool allows the professional to define the real need to use infant formula, either as a complementary or exclusive form ( Figure 2 ).

Perhaps the greatest contribution of this study is the development of this flowchart to support decision making regarding the use of infant formula. To the best of our knowledge, there is still no similar tool developed to meet the Brazilian reality. The tool can be adopted - in its entirety or after adjustments to the local context - in health units, regardless of the level of care, as a way to avoid errors in prescribing infant formula and to favor the implementation of the national breastfeeding policy.

Furthermore, the fact that retrospective data was used was considered a limiting factor of this study, which made it difficult to identify all the necessary information in the medical records.

## CONCLUSION

The high rates of infant formula prescription in the studied population were a concern, since breastfeeding is far below the national averages and because this population was assisted by health professionals of the Public Health System. Moreover, the patients cared for were at high medical and nutritional risk, according to the criteria for referral to the center, and should not be deprived of the health, emotional, social, and economic benefits of breastfeeding. Thus, the research team considers important the adoption of the decision-making flowchart regarding the prescription of infant formula, and the inclusion of the training of prescribers at different levels of health care in the strategies to encourage breastfeeding.

Unnecessary expenses are emphasized, due to the high cost to the government because of the indiscriminate use of infant formulas. Thus, it is important to reevaluate public and private policies that prioritize the offer of infant formulas, much more expensive and with fewer benefits for the mother and infant.
