# Supplementary material for: Breastfeeding versus free distribution of infant formulas by the Public Health System
Source: Einstein (Sao Paulo). 2021 Nov 3;19:eAO6451. doi: 10.31744/einstein_journal/2021AO6451 (PMC8577314; doi:10.31744/einstein_journal/2021AO6451)
Supplement: Translation

# Translation: Aleitamento materno *versus* distribuição gratuita de fórmulas infantis pelo Sistema Único de Saúde

**Authors:** Flávia Galvão Cândido, Brunnella Alcântara Chagas de Freitas, Rita de Cássia Santos Soares, Jersica Martins Bittencourt, Daniela Neves Ribeiro, Dayane de Castro Morais, Camilla de Freitas Niquine, Sarah Aparecida Vieira Ribeiro, Raquel Maria Amaral Araújo, Bruna Romano Zucchetto, Taimã de Castro Carvalho, Isabela Carvalho Rezende

**Affiliations:** 1 ViçosaMG Brasil Centro Estadual de Atenção Especializada, Viçosa, MG, Brasil.; 2 ViçosaMG Brasil Universidade Federal de Viçosa, Viçosa, MG, Brasil.

**Keywords:** Aleitamento materno, Fórmulas infantis, Prescrições, Sistema Único de Saúde

## Abstract

**Objetivo:**

Caracterizar a situação de aleitamento materno e da adequação de prescrição de fórmulas infantis a lactentes assistidos por um programa de Atenção Secundária do Sistema Único de Saúde.

**Métodos:**

Trata-se de estudo transversal pela análise de prontuários de 350 lactentes de zero a 6 meses, acompanhados entre fevereiro a abril de 2019.

**Resultados:**

A possibilidade de aleitamento materno esteve presente em 97,0% das mães, e nenhum lactente apresentou condição médica aceitável para a proscrição do aleitamento materno. Apesar disso, apenas 47,2% dos casos estavam em aleitamento materno exclusivo antes do encaminhamento ao programa. Em relação aos motivos da introdução das fórmulas infantis, a complementação ao leite materno foi o mais presente (75,8%), seguido da volta da mãe ao mercado de trabalho (20,1%). As taxas gerais de inadequação dentre os prescritos foram de 65% antes da chegada ao programa, passando para 69% (fórmulas padrão) e 80% (fórmulas para fins especiais) durante o acompanhamento.

**Conclusão:**

A baixa taxa de aleitamento materno exclusivo e a prescrição indiscriminada de fórmulas infantis são preocupantes por causar prejuízos à saúde maternoinfantil e financeira do Sistema Único de Saúde.

## INTRODUÇÃO

O leite materno é, indubitavelmente, o melhor alimento para o recém-nascido e deve ser mantido exclusivamente até os 6 meses de vida.^( 1 )^Estima-se que se a amamentação fosse ampliada para níveis quase universais, cerca de 820 mil vidas de crianças seriam salvas a cada ano.^( 2 )^Globalmente, apenas 40% das crianças com menos de 6 meses de idade recebem aleitamento materno exclusivo (AME). No Brasil, apesar da melhoria dos indicadores de aleitamento materno, as pesquisas revelam tendência à estabilização dos índices nos últimos anos, com apenas 36,6% dos bebês recebendo AME até os 6 meses de vida.^( 3 )^

O Ministério da Saúde tem adotado iniciativas importantes visando à melhoria dos indicadores nacionais de AME.^( 1 – 4 )^No entanto, concomitante a esses esforços e justificando-se pela necessidade de proteger bebês em situação de risco nutricional, muitos programas têm sido desenvolvidos nas diversas regiões brasileiras para promover a distribuição gratuita pelo Sistema Único de Saúde (SUS) de fórmulas infantis como substitutas do leite materno.^( 5 )^

As fórmulas infantis são desenvolvidas para se assemelharem ao leite materno e podem ser prescritas em condições médicas e/ou nutricionais bem específicas, na maioria das vezes, por um período limitado de tempo.^( 6 )^No entanto, a composição das fórmulas infantis não se iguala às propriedades fisiológicas e nutricionais do leite humano, além de impactar negativamente no sucesso e na duração do aleitamento materno.^( 5 )^O Ministério da Saúde ressalta como sendo “absolutamente condenável a distribuição em larga escala destes produtos, em especial em serviços de saúde”.^( 5 )^Excluindo-se os casos em que existem razões médicas aceitáveis para o uso de leites ou fórmulas infantis, a prescrição desses produtos para bebês que não necessitam desses alimentos deve ser considerada inapropriada.^( 7 )^

Outro aspecto a ser considerado da distribuição refere-se às repercussões econômicas do uso de fórmulas infantis. Segundo a lei federal 8.080/1990, a alimentação é caracterizada como um dos fatores condicionantes da saúde (Art. 3º, *caput* ) e estabelece a vigilância nutricional e orientação alimentar (Art. 6º) como atribuições específicas do SUS. Assim, nos casos em que a alimentação assume *status* de fármaco, como é o caso das fórmulas infantis, cabe ao setor público o fornecimento das dietas, de acordo com os princípios e as normas do SUS. Segundo o Art. 6º “ São direitos sociais a educação, a saúde, a alimentação, o trabalho, a moradia, o transporte, o lazer, a segurança, a previdência social, a proteção à maternidade e à infância, a assistência aos desamparados, na forma desta Constituição.^( 8 )^

Diante do considerável número de prescrições de fórmulas infantis sem indicações bem estabelecidas, tornou-se urgente o estabelecimento de critérios para racionalizar o acesso e propor um fluxo possível, equitativo e igualitário para a dispensação, otimizando os recursos públicos gastos com a compra das fórmulas infantis.^( 5 )^

## OBJETIVO

Caracterizar a situação de aleitamento materno e da adequação de prescrição de fórmulas infantis a lactentes assistidos por um programa de Atenção Secundária do Sistema Único de Saúde.

## MÉTODOS

### Delineamento

Trata-se de um estudo transversal quantitativo exploratório realizado no Centro Estadual de Atenção Especializada (CEAE) do município de Viçosa (MG). Os dados foram obtidos a partir de prontuários de pacientes de zero e 6 meses encaminhados ao CEAE, atendidas pelo Programa Leite para Todos entre fevereiro a abril de 2019.

Foram excluídos os pacientes que não contemplavam os critérios de inclusão de lactentes do CEAE descritos na tabela 1 , apresentavam escassez de dados no prontuário sobre o tipo de aleitamento, não foram atendidos pelo serviço de nutrição do centro, com ausência de dados retrospectivos confiáveis nos prontuários, mães adolescentes (<16 anos), pacientes que realizaram o primeiro atendimento com nutrição e/ou iniciaram o acompanhamento após o período estabelecido.

**Tabela 1:** Critérios para inclusão de pacientes neonatologia/pediatria

Neonatologia
Prematuridade ≤34 semanas ou peso ao nascer ≤1.800g
Recém-nascidos prematuros ou não que tiveram sofrimento fetal agudo: escore de Apgar < +7 no quinto minuto de vida
Egressos de internação em unidades neonatais de cuidados progressivos (unidade de terapia intensiva neonatal, unidade de cuidado intermediário convencional, unidade de cuidado intermediário canguru)
Doenças infecciosas de transmissão perinatal: HIV, Síndrome TORCHS e Zika
Patologias classificadas como raras que necessitem de manejo por equipe multiprofissional, incluindo necessidade de medicação ou dieta específica
Prematuros com doença pulmonar crônica
Recém-nascido com alterações do desenvolvimento neuropsicomotor
Recém-nascido com alterações hematológicas crônicas

Pediatria
Crianças egressas da UTI pediátrica
Crianças com doenças gastrintestinais crônicas com repercussão nutricional (diarreia crônica, doença celíaca, atresia de vias biliares, cirroses, alergias alimentares etc.)
Crianças com imunodeficiências congênitas
Crianças com alterações renais crônicas (ITU de repetição, nefroses, hematúrias etc.)
Crianças com problemas oncológicos
Crianças com distúrbios psiquiátricos e/ou TDAH
Crianças com diabetes (primeira consulta, sendo as outras na Atenção Primária)
Crianças portadoras de cardiopatias congênitas e hipertensão arterial
Patologias classificadas como raras que necessitem manejo por equipe multiprofissional, incluindo o uso de medicação e/ou dieta específica

TORCHS: toxoplasmose, rubéola, citomegalovirus, herpes simples, sífilis; UTI: unidade de terapia intensivo; ITU: infecção do trato urinário; TDAH: transtorno do défice de atenção com hiperatividade.

### Planilhas de coleta de dados

Durante a elaboração da planilha de coleta de dados, foram selecionados aleatoriamente 20 questionários para serem avaliados quanto a tipo, qualidade e relevância das informações registradas em relação à proposta do programa. A partir de então, de acordo com as experiências dos profissionais/autores e da revisão dos prontuários incluídos na análise, selecionaram-se as questões importantes a serem respondidas para a caracterização do problema e a formulação de soluções. A seleção das variáveis de nível socioeconômico e demográfico, de saúde geral e de nutrição baseou-se na capacidade de elas responderem às perguntas e permitirem a elaboração de soluções, bem como na importância epidemiológica que elas apresentavam, indicada pela literatura científica sobre a temática.^( 9 )^

As variáveis socioeconômicas e demográficas foram sexo, cidade de origem, escolaridade materna, renda familiar e unidade de encaminhamento ao CEAE. As variáveis de saúde geral foram número de gestações anteriores, duração do acompanhamento nutricional no CEAE e presença de condições/patologias maternas ou do bebê que inviabilizaram ou comprometeram o aleitamento materno. As variáveis de nutrição foram tipo de aleitamento antes e durante o acompanhamento, prescrição de fórmula infantil (padrão ou com finalidades especiais), adequação da prescrição de fórmulas e duração do aleitamento materno após a prescrição das fórmulas.

### Análise dos dados

Para a descrição das condições e das patologias que inviabilizaram ou comprometeram o aleitamento materno, bem como para a avaliação da adequação das prescrições das fórmulas infantis, foram utilizadas as Razões Médicas Aceitáveis para Uso de Substitutos do Leite Materno, que são casos de sorologia positiva para HIV, dependência química, em tratamento quimioterápico, uso de medicamentos e etilismo.^( 7 )^A escolha desse documento como critério de avaliação deu-se por sua ampla aceitação por profissionais e gestores de saúde e pela objetividade do conteúdo, que possibilita a não subjetividade da avaliação. O uso de fórmulas infantis por mudanças de rotina da mãe ou do bebê, como frequentar creches ou retorno da mãe ao mercado de trabalho, também foi considerado inadequado, por não constar no documento e pela possibilidade de ordenha do leite para oferecimento e/ou ida às creches pelas mães para aleitamento materno.

A análise dos questionários foi realizada por quatro avaliadores independentes previamente capacitados. Os casos duvidosos foram separados e discutidos em conjunto, de modo a padronizar o máximo possível a análise. A ausência de consenso foi interpretada como dado não disponível.

Foram adotadas a idade cronológica em pacientes nascidos a termo (37 a 42 semanas de gestação) e a idade corrigida (IC) em pacientes nascidos prematuros (com menos de 37 semanas de gestação), para a determinação do período de acompanhamento da evolução nutricional (zero a seis meses) e do período de aleitamento materno.^( 10 )^Nos casos de pacientes prematuros em uso exclusivo de fórmula infantil, o período de avaliação foi realizado até a introdução da alimentação complementar, devido à possibilidade de introdução precoce da alimentação complementar nesses casos, nunca ultra-passando o limite inferior de 4 meses de IC.^( 2 )^

A descontinuidade do aleitamento materno foi considerada nos seguintes casos: a mãe parou de amamentar, os lactentes estavam em uso apenas de fórmula infantil e/ou o aleitamento materno parou de ser mencionado nas fichas de evolução nutricional do paciente. Nos casos de recebimento de alta no CEAE por bebês em AME com idade inferior a 6 meses e que foram devidamente orientados e incentivados a manter a AME, foi considerada a continuação do AME até os 6 meses.

Foi considerado dado faltante sobre possibilidade de aleitamento materno, pela mãe, aquelas cujos bebês foram encaminhados em uso exclusivo se fórmula infantil e cujos prontuários não continham informações precisas sobre as condições de lactação maternas. Foram considerados lactentes aptos para aleitamento materno aqueles encaminhados em ame ou misto (aleitamento materno mais fórmula infantil) sem a descrição de condições do lactente que inviabilizam a lactação, ou nos casos cujas condições foram corrigidas antes do encaminhamento.

Foram adotados apenas aspectos qualitativos para a avaliação da adequação da prescrição de fórmulas infantis. As fórmulas infantis especiais foram consideradas como qualquer formulação que não fosse padrão ou de partida (fórmulas tipo 1).

Foram consideradas inadequadas as prescrições que não atenderam às razões médicas aceitáveis para a prescrição de fórmula infantil acrescidas de outras condições/patologias com potencial de dificultar o aleitamento materno, como a escabiose, a mastite, a pouca aceitação do aleitamento materno, o relato de baixa produção de leite, o óbito materno e/ou a impossibili-dade de convivência com a mãe. A adequação e a inadequação das prescrições foram avaliadas em dois momentos distintos: no ato do encaminhamento ao CEAE e durante o acompanhamento no centro. Considerou-se adequada a prescrição de fórmulas hipercalóricas apenas nos casos de patologias do lactente com necessi-dade de restrição hídrica.

Para os cálculos das repercussões econômicas, utilizaram-se como base as quantidades médias de fórmulas recomendadas pelo próprio fabricante a lactentes de zero a 6 meses e a duração informada do aleitamento materno, além dos valores médios de fórmulas padrão ou para fins especiais disponíveis em uma grande rede varejista. As quantidades de fórmulas recomendadas para suprir as necessidades do lactente foram integral-mente convertidas em valores monetários para os lactentes em uso exclusivo de fórmula. Para os lactentes em uso complementar de fórmula infantil, a conversão foi realizada para atingir 50% das necessidades. Os cálculos foram feitos de acordo com o tipo de fórmula utilizada para cada lactente. Consideraram-se na contabilização das repercussões econômicas negativas apenas aqueles casos em que a prescrição foi considerada inadequada de acordo com os critérios do estudo.

Após a coleta, os dados foram tabulados em uma planilha do programa Excel, e a análise estatística foi conduzida com o auxílio do software (SPSS), versão 17.0 (SPSS Inc., Chicago, Estados Unidos).

As variáveis foram testadas quanto à normalidade da distribuição dos dados pelo teste de Kolmogorov-Smirnov e descritas em frequência absoluta e relativa. Foi realizado o teste de Mann-Whitney para comparar os dados quanto ao tempo de aleitamento materno. A análise de correlação de Spearman foi usada para variáveis contínuas. Para todas essas análises, adotou-se alfa de 5%.

Este trabalho foi aprovado pelo Comitê de Ética em Pesquisa, recebendo CAAE: 16796619.1.0000.5153, parecer 3.515.075.

## RESULTADOS

O número total de prontuários avaliados foi 495, sendo incluídos 350 a partir dos critérios de seleção adotados ( Figura 1 ). A possibilidade de aleitamento materno esteve presente em 97% das mães avaliadas, e nenhum lactente apresentou condição médica aceitável para a proscrição do aleitamento materno. Todavia, apenas 47,2% dos casos estavam em AME antes do encaminhamento ao CEAE, com a redução dessa taxa para apenas 28,0% durante o acompanhamento no CEAE. Somente 10,9% dos neonatos que chegaram ao CEAE em uso complementar de fórmula infantil passaram a receber AME; 41,1% permanecem com o uso complementar de fórmula infantil e 48,0% passaram a usar fórmula infantil de maneira exclusiva.

**Figura 1:** Fluxograma amostral de acordo com o setor de encaminhamento e os estágios da caracterização do problema

Verificou-se que 92% (n=149) dos pacientes faziam uso de fórmula infantil antes do encaminhamento ao CEAE e continuaram consumindo após o acompanhamento no centro, enquanto 54% (n=94) dos que não utilizavam antes do início do acompanhamento continuaram sem essa prescrição. Assim, houve associação entre utilização de fórmula infantil anterior ao encaminhamento ao CEAE e continuidade da prática após o acompanhamento no centro, demonstrando que a introdução precoce de fórmulas dificulta a retomada do AME. Entre os prontuários que continham informações sobre o motivo da introdução da fórmula infantil (n=124), a complementação ao leite materno foi o mais presente (75,8%), seguido pela volta da mãe ao mercado de trabalho (20,1%).

O aleitamento materno não teve associação significativa com as variáveis paridade, renda e escolaridade. As variáveis gestação gemelar, paridade, renda e escolaridade não tiveram efeito sobre o tempo de aleitamento materno. O tempo de aleitamento materno apresentou mediana de 120 dias (zero a 1.260 dias), enquanto o tempo de acompanhamento no centro foi de 270 dias (15 a 1.740 dias). Houve correlação positiva entre essas variáveis (r=0,176), indicando que o aumento do tempo de acompanhamento no centro ocasionava aumento do tempo de aleitamento materno das crianças, mas a gemelaridade associou-se negativamente com AME, como mostra a tabela 2 .

**Tabela 2:** Relação entre aleitamento materno exclusivo e características fisiológicas e socioeconômicas das mães avaliadas

Variáveis		Aleitamento materno exclusivo		Valor de p *
		Sim	Não	
Gestação gemelar				
	Sim	13 (30,2)	30 (69,8)	0,015
	Não	149 (50,0)	149 (50,0)	
Paridade				
	Multípara	74 (43,0)	98 (57,0)	0,74
	Primípara	85 (52,8)	76 (47,2)	
Renda, salários mínimos †				
	≤3	87 (44,6)	108 (55,4)	0,882
	>3	13 (52,0)	12 (48,0)	
Escolaridade				
	≤Ensino medio incompleto	47 (42,7)	63 (57,3)	0,346
	≥Ensino medio completo	52 (43,7)	67 (56,3)	

Resultados expressos por n (%).

* Teste do χ^2^de Pearson.

† Salario minimo considerado R$ 1.006,00.

As projeções das repercussões econômicas estimadas dos custos com a aquisição de fórmulas infantis e especiais que poderia ser evitada foram, respectivamente, R$ 80.814,00 e R$ 55.942,14. As estimativas de custos totais com fórmulas especiais foram de R$ 85.427,12 para atender 51 bebês, enquanto os custos com fórmulas padrão foram de R$ 110.568,82 para atender 187 bebês. Devido à grande inadequação na prescrição de fórmulas infantis, os custos considerados evitáveis excederam os necessários em 130,9% para todas as fórmulas, em 171,6% para fórmula padrão e em 89,7% para as fórmulas especiais.

## DISCUSSÃO

Este estudo evidenciou baixas taxas de AME, bem como excesso de prescrição e distribuição gratuita de fórmulas infantis em lactentes menores de 6 meses assistidos por um programa de Atenção Secundária do SUS. As taxas de AME encontram-se ainda inferiores à média nacional (28,0% *versus* 36,6%), que já é considerada baixa por órgãos de renome internacional.^( 3 )^Sabe-se que o leite materno transfere proteção imunológica específica da mãe para o próprio filho durante a lactação,^( 11 )^sendo capaz de beneficiar a função intestinal e proteger a criança contra pneumonias, infecções de ouvido e alergias, além de propiciar melhor desenvolvimento do sistema nervoso.^( 1 , 12 )^

As baixas taxas de AME observadas nesta pesquisa ocorreram na ausência de fatores restritivos e impeditivos ligados à mãe e ao bebê na maioria da amostra. A única razão médica aceitável ligada ao lactente que, de fato, implica na impossibilidade de consumo do leite materno é a presença de erros inatos do metabolismo, como a galactosemia e a leucinose,^( 11 )^o que não ocorreu em nenhum paciente avaliado.

O uso de fórmulas infantis tem sido associado negativamente a maiores prevalências de obesidade, diabetes, hipertensão arterial e vários tipos de câncer na idade adulta, sendo o aleitamento materno considerado fator protetor, além de estar relacionado com desmame precoce.^( 2 , 13 )^Excluindo-se casos em que há razões médicas aceitáveis para o uso de leites ou fórmulas infantis,^( 7 )^a prescrição desses produtos para crianças que não necessitam desses alimentos deve ser considerada inapropriada.

O uso complementar de fórmula infantil deve ser avaliado de maneira criteriosa, pois a prescrição de substitutos do leite materno nas maternidades pode encorajar as mães a fazerem o mesmo ao retornarem para casa, acreditando que são incapazes de produzir leite em quantidade suficiente para o recém-nascido.^( 14 )^

Os resultados apresentados ressaltam a necessidade urgente de avaliação da política atual de prescrição e distribuição de fórmulas infantis, que parece ser demasiada e incoerente com políticas nacionais de promoção do aleitamento materno. Ainda, demonstram a necessidade de uma melhor articulação com os demais níveis de atenção (primários e terciários), já que o problema foi evidenciado antes mesmo da chegada do paciente para assistência. Tais resultados, além de soarem como um sinal de alerta, pelas questões de saúde, possuem implicações financeiras para a gestão pública também relevantes.

A unidade de Atenção Secundária estudada adota a política de distribuição gratuita de fórmulas infantis mediante apresentação de receituário preenchido por nutricionista. A liberação é feita pelos municípios cadastrados, situação similar à da maioria das unidades de Atenção Secundária brasileiras. Os cálculos neste trabalho foram realizados para atender à demanda das prescrições feitas apenas para os lactentes que possuíam prontuários ativos durante fevereiro a abril de 2019, considerando as necessidades nutricionais do zero aos 6 meses apenas.

Segundo as estimativas desta pesquisa, manter uma criança em aleitamento artificial exclusivo com fórmulas padrão de 0 aos 6 meses de idade pode custar, em média, R$ 1.000,00 para o município pelo período de 6 meses. Esse valor pode aumentar para até cerca de R$ 9.800,00 no mesmo intervalo de tempo, caso a prescrição seja feita para fórmulas especializadas. Uma vez que as unidades de Atenção Secundárias recebem novos pacientes ao longo do ano e que é usual a manutenção das fórmulas infantis até pelo menos os 12 meses de vida, as projeções anuais podem ser bem maiores. Tendo em vista a importância do uso racional dos recursos públicos, por seu caráter limitado, a prescrição coerente de fórmulas infantis assume caráter de responsabilidade pública, por permitir destinar recursos escassos a quem realmente precisa.

A partir dos resultados do presente estudo, foi elaborado um fluxograma de auxílio à tomada de decisão quanto à prescrição de fórmula infantil, baseado nas recomendações da Organização Mundial da Saúde (OMS),^( 7 )^que pode ser usado por profissionais de saúde, como pediatras e nutricionistas, durante o atendimento de lactentes. Esse instrumento permite, por meio da avaliação das condições maternas e da criança, que o profissional defina a real necessidade de utilização da fórmula infantil, seja de forma complementar ou exclusiva ( Figura 2 ).

**Figura 2:** Fluxograma de decisão para prescrição de fórmulas infantis a lactentes de zero a 6 meses

Talvez a maior contribuição deste estudo seja a elaboração deste fluxograma para embasar a tomada de decisão quanto ao uso de fórmula infantil. Até o que se sabe, ainda não há um instrumento semelhante desenvolvido para atender à realidade brasileira. O instrumento pode ser adotado – na íntegra ou após adaptações à realidade local – em unidades de saúde, independentemente do nível de atenção, como forma de evitar erros de prescrição de fórmula infantil e favorecer a implementação da política nacional de aleitamento materno.

Ademais, foi considerado um fator limitante deste estudo o fato de terem sido utilizado dados retrospectivos, o que dificultou a identificação de todas as informações necessárias nos prontuários.

## CONCLUSÃO

As altas taxas de prescrição de fórmulas infantis na população estudada foram preocupantes, uma vez que a amamentação se encontra muito abaixo das médias nacionais e por se tratar de pessoas assistidas por profissionais da saúde do Sistema Único de Saúde. Além disso, os pacientes assistidos possuíam alto risco médico e nutricional, por critérios de encaminhamento ao centro, e não deveriam ser privados dos benefícios à saúde, afetivos, sociais e econômicos do aleitamento materno. Desse modo, a equipe de pesquisa considera importante a adoção do fluxograma de tomada de decisão quanto à prescrição de fórmula infantil e a inclusão da capacitação dos prescritores dos diversos níveis de Atenção à Saúde nas estratégias para incentivo ao aleitamento materno.

Ressaltam-se os gastos desnecessários, devido ao elevado custo para o poder público por conta do uso indiscriminado de fórmulas infantis, tornando-se importante reavaliar as políticas públicas e privadas que priorizam o oferecimento de fórmulas infantis, muito mais onerosas e com menos benefícios para a lactante e o lactente.
